# Remnant cholesterol, C-reactive protein, cholesterol-lowering medication, and cardiovascular outcome: novel findings from UK Biobank

**DOI:** 10.3389/fnut.2026.1920077

**Published:** 2026-10-05

**Authors:** Jing Chen, Xuan Song, Jian-Jun Li, Li-Chan Tao

**Affiliations:** 1 Department of Cardiology, The Third Affiliated Hospital of Soochow University, Changzhou, China; 2 Department of Endocrinology, The Third Affiliated Hospital of Soochow University, Changzhou, China; 3 Department of Cardiology, Cardiometabolic Center, State Key Laboratory of Cardiovascular Diseases, Fu Wai Hospital, National Center for Cardiovascular Diseases, Chinese Academy of Medical Sciences and Peking Union Medical College, Beijing, China

**Keywords:** cardiovascular diseases, cholesterol-lowering medication, C-reactive protein, major adverse cardiovascular events, remnant cholesterol

## Abstract

**Background:**

Despite substantial reductions in cardiovascular events with intensive LDL-C lowering, significant residual risk persists. Remnant cholesterol (RC) and C-reactive protein (CRP) reflect lipid dysregulation and inflammation, respectively, and may synergistically promote atherosclerosis. However, large-scale cohort evidence on their combined impact, particularly the quantitative interaction between the two and the modifying role of cholesterol-lowering therapy, remains limited.

**Methods:**

This study included 376,817 participants with a median follow-up of 13.4 years. Cox proportional hazards regression models were used to evaluate the individual and joint associations of RC and CRP with major adverse cardiovascular events (MACE) risk. Stratified analyses by cholesterol-lowering therapy were further conducted to explore its modifying effect on the combined risk.

**Results:**

Both RC (≥0.8 mmol/L) and CRP (≥2 mg/L) were independently associated with MACE risk, with adjusted hazard ratios (HR) of 1.074 [95% confidence interval (CI): 1.024–1.126] and 1.168 (95% CI: 1.129–1.207), respectively. Concurrent elevation of both biomarkers conferred the highest risk (HR = 1.239, 95% CI: 1.167–1.316), and a significant positive multiplicative interaction was observed between RC and CRP. Stratified analyses suggested effect modification by cholesterol-lowering medication: the association between isolated RC elevation and MACE was attenuated in medication users, whereas risks remained significant for isolated CRP elevation and combined elevation of both markers.

**Conclusion:**

Concurrent elevation of RC and CRP yields the highest MACE risk with a positive additive interaction. Cholesterol-lowering therapy reduces RC-related risk but not inflammation-driven risk in isolated CRP elevation. These findings support combined assessment of both biomarkers for personalized cardiovascular risk stratification.

## Graphical Abstract

**Figure:**
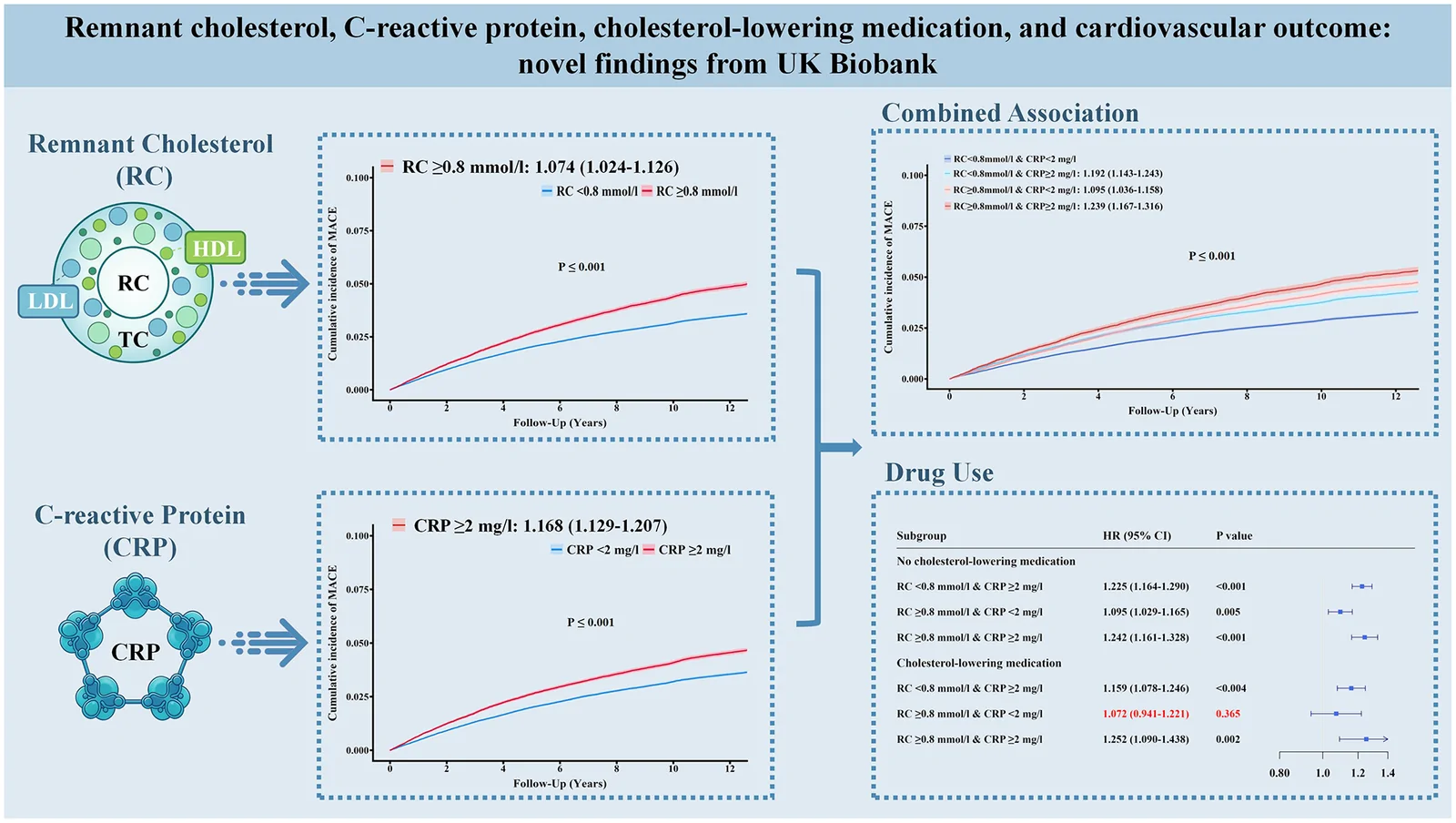
The left panels and the top right panel show the independent and joint associations of RC and CRP. Both RC ≥0.8 mmol/L and CRP ≥2 mg/L were significantly associated with an increased risk of MACE. In the joint analysis, compared with the reference group (RC < 0.8 mmol/L and CRP < 2 mg/L), the group with RC ≥0.8 mmol/L and CRP ≥2 mg/L had the highest risk of MACE. The bottom right panel shows the stratified analysis by cholesterol-lowering medication use. Among participants not using cholesterol-lowering medication, all three combined exposure groups were significantly associated with MACE risk. Among participants using cholesterol-lowering medication, the other two groups remained statistically significant except for the group with RC ≥0.8 mmol/L and CRP < 2 mg/L. Models were adjusted for age, sex, smoking, drinking, ethnicity, diet score, physical activity, LDL-C, TG, diabetes, hypertension, antihypertensive agents, and hypoglycemic agents. P values represent the significance compared with the reference group; P ≤ 0.05 was considered statistically significant. CI, confidence interval; CRP, C-reactive protein; HR, hazard ratio; LDL-C, low-density lipoprotein cholesterol; MACE, major adverse cardiovascular events; RC, remnant cholesterol; TG, triglycerides.

## Introduction

Cardiovascular disease (CVD) remains the leading cause of death and disability worldwide. Despite substantial advances in cholesterol-lowering therapies, a significant residual cardiovascular risk persists, suggesting that pathways beyond traditional lipid targets contribute to atherogenesis. Atherosclerosis, the pathological foundation of most cardiovascular events, involves a complex interplay between lipid dysregulation and inflammatory responses (1, 2). Low-density lipoprotein cholesterol (LDL-C) has been firmly established as a causal risk factor and serves as the primary target for clinical intervention (3). However, a considerable proportion of patients still experience cardiovascular events despite intensive LDL-C lowering, underscoring the need to identify and address residual risk mechanisms beyond LDL-C.

In recent years, remnant cholesterol (RC), the cholesterol content within triglyceride-rich lipoproteins, has been increasingly recognized as an independent atherogenic component (4–6). A recent dose-response systematic review and meta-analysis further demonstrated a linear positive association between RC levels and cardiovascular events in patients with diabetes, providing robust epidemiological evidence for the causal role of RC in atherogenesis (7). RC particles readily deposit within the arterial wall, promote foam cell formation, and accelerate plaque progression (8, 9). Large-scale epidemiological studies have confirmed that elevated RC levels are independently associated with myocardial infarction (MI) (10), ischemic stroke (11), and coronary heart disease (12), even after adjustment for LDL-C. Beyond lipid-driven risk, systemic low-grade inflammation represents another important contributor to residual cardiovascular risk. C-reactive protein (CRP), a classic biomarker of inflammation, has been consistently shown to predict future cardiovascular events and provide incremental value beyond traditional risk factors (13).

Of note, RC and CRP are not independent risk factors. Cholesterol overload can activate inflammatory cascades, while inflammatory cytokines in turn impair macrophage cholesterol efflux, creating a bidirectional vicious cycle (14–16). This interplay suggests that assessing RC or CRP alone may underestimate an individual's true cardiovascular risk, and that combined evaluation of both biomarkers may more comprehensively capture the underlying pathophysiological burden.

However, large-scale prospective evidence on the combined effect of RC and CRP in predicting cardiovascular risk remains limited, and whether an interaction exists between these two biomarkers is still unclear. We therefore hypothesize that concomitant elevation of RC and CRP may represent a high-risk phenotype, and that combined assessment offers superior predictive value compared to either marker alone. To test this hypothesis, this study aims to systematically evaluate the association of combined RC and CRP levels with the risk of major adverse cardiovascular events (MACE), including non-fatal MI, non-fatal stroke, and cardiovascular death, using data from the large, prospective UK Biobank (UKB) cohort, and to further explore potential interactions between RC and CRP, thereby providing novel evidence for risk stratification and precision prevention in CVD.

## Methods

### Data sources and study population

This study was based on data from the UKB, a large-scale prospective cohort study integrating genetic, phenotypic, and health-related records from participants across the United Kingdom. Study participants were recruited from 22 clinical research centers between 2006 and 2010. At baseline, all participants completed structured touchscreen questionnaires and underwent comprehensive assessments covering sociodemographic characteristics, lifestyle, health status, and physical examinations. Long-term follow-up information on hospitalizations, clinical outcomes, and deaths was obtained through linkage to primary care, hospital electronic medical records, and death registration databases. Endpoint events were defined according to the International Classification of Diseases, Tenth Revision (ICD-10). This study was approved by the Northwest Multi-Center Research Ethics Committee in the UK, and all participants provided written informed consent.

During the baseline survey period from 2006 to 2010, a total of 502,301 participants were included in the study. To evaluate the combined effect of RC and CRP on MACE, we first excluded 56,742 individuals who had prevalent cardiovascular events at baseline, and subsequently further excluded 65,775 participants with missing data on either RC or CRP. When total cholesterol (TC) levels are markedly elevated, a large number of very low-density lipoprotein (VLDL) particles accumulate in the blood, resulting in abnormal particle size and density. This interferes with the direct measurement or indirect estimation of LDL-C, leading to substantial deviations in LDL-C values (17). Meanwhile, when TC levels are elevated, VLDL cholesterol concentrations increase substantially. If RC is still calculated using the formula *RC* = *TC*−*HDL*−*C*−*LDL*−*C*, interference from VLDL and inaccuracies in LDL-C measurement may introduce systematic errors, making it difficult to accurately reflect the true RC burden (18). Therefore, 2,967 individuals with TG ≥5.7 mmol/L were excluded from the study. The final analytic sample comprised 376,817 participants. With the aim of further controlling for the influence of cholesterol-lowering therapy, we proceeded to exclude 3,129 participants whose records on such medication were incomplete, culminating in the inclusion of 373,688 participants in the final analysis. The participant selection process is summarized in the study flowchart (Figure 1).

**Figure 1:**
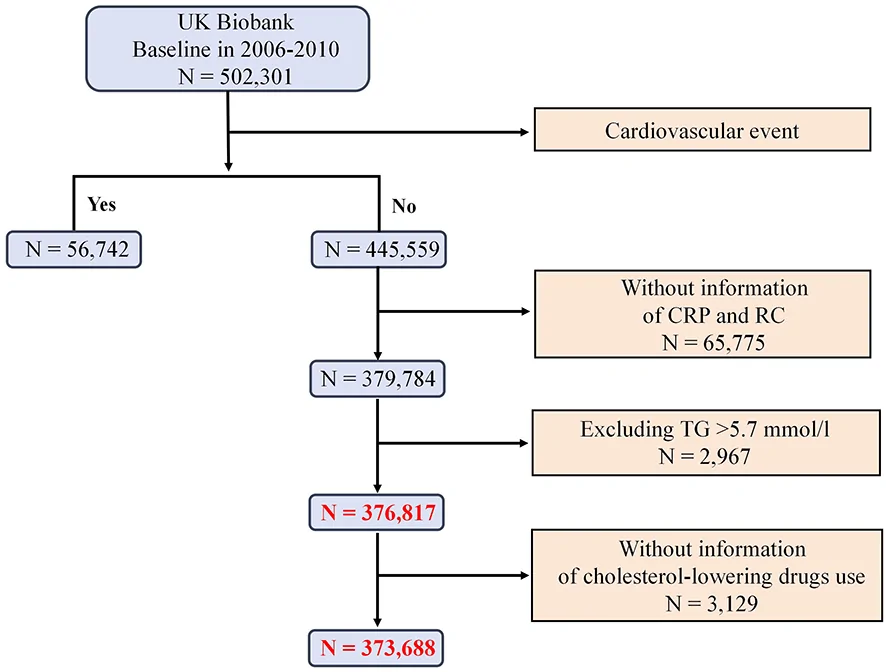
Flowchart of study population selection from the UK Biobank cohort. The UK Biobank baseline survey carried out between 2006 and 2010 initially recruited 502,301 participants. We excluded 56,742 individuals who had a history of cardiovascular disease at the time of enrolment, which left us with 445,559 people. From this group, we removed 65,775 participants because they had missing values for either CRP or RC, reducing the sample to 379,784. We then dropped 2,967 participants whose triglyceride levels exceeded 5.7 mmol/L, and that gave us a cohort of 376,817 individuals, which we labeled Cohort A. Finally, we excluded a further 3,129 participants who did not have information on whether they were taking cholesterol-lowering drugs. After that final exclusion, we obtained our final analytical sample, Cohort B, consisting of 373,688 participants.

### Assessment of RC and CRP

TC, HDL-C, and direct LDL-C were measured enzymatically using a Beckman Coulter AU5800 automatic biochemical analyzer. TC was determined by the cholesterol oxidase-peroxidase (CHO-POD) method, HDL-C by the immune-inhibition method, and LDL-C by the enzyme protection method. All measurements were performed on non-fasting serum samples. RC was calculated using the formula RC = TC–HDL–C–LDL–C with LDL based on the directly measured value (19). CRP was measured by latex-enhanced immunoturbidimetry on the same Beckman Coulter AU5800 analyzer, also using non-fasting serum samples.

### Outcomes

The primary outcome of this study was the first occurrence of MACE, defined as a composite endpoint of non-fatal MI, non-fatal stroke, and cardiovascular death (20). Outcome events were identified through linkage with hospital inpatient data and death registration systems and were confirmed based on corresponding ICD-10 codes (Supplementary Table 10). Follow-up duration was calculated from the baseline assessment date to the date of first MACE occurrence, death, loss to follow-up, or the study end date, whichever occurred first.

### Demographic characteristics

Potential confounding factors were obtained through structured questionnaires and interviews, primarily including sociodemographic characteristics (age, sex, race), lifestyle factors (current smoking, current drinking), physical activity level, dietary score, and history of chronic diseases. Laboratory indicators included LDL-C and TG. Physical activity level was quantified in metabolic equivalents (METs) and categorized into three levels based on total weekly MET-hours: low (< 10 MET-h/week), moderate (10–50 MET-h/week), and high (>50 MET-h/week). The healthy diet score was constructed based on five healthy dietary behaviors: daily vegetable intake ≥4 servings, daily fruit intake ≥3 servings, weekly fish intake ≥2 times, weekly processed red meat intake ≤ 2 times, and weekly processed meat intake ≤ 2 times. Each behavior was assigned 1 point, yielding a total score ranging from 0 to 5. CVD events were identified based on ICD-10 codes I20, I21, I22, I23, I24, I25, I46, I60, I61, I63, and I64. Medical histories of diabetes and hypertension were extracted from hospital inpatient records using the corresponding ICD-10 codes (Supplementary Table 10). Antihypertensive and cholesterol-lowering medication use was determined based on UKB field code 6177 and 6153, whereas hypoglycemic agent use was derived from self-reported medication information collected at the baseline visit (Supplementary Table 11).

### Individual associations of RC and CRP with MACE

The Cox proportional hazards regression model was used to evaluate the associations of CRP and RC levels with MACE events. CRP levels were categorized as < 2 and ≥2 mg/L, and RC levels as < 0.8 and ≥0.8 mmol/L, with the lower level group serving as the reference in each analysis. Hazard ratios (HR) and 95% confidence intervals (CI) were calculated for each model. The cumulative incidence of MACE events across different CRP and RC groups was estimated using the Kaplan–Meier method, and differences between groups were compared using the log-rank test.

### Association of the combined effect of RC and CRP with MACE risk

To evaluate the combined effect of RC and CRP on MACE, participants were divided into four groups based on the clinical cutoffs: the low-low group (RC < 0.8 mmol/L and CRP < 2 mg/L), the single elevated CRP group (RC < 0.8 mmol/L and CRP ≥ 2 mg/L), the single elevated RC group (RC ≥ 0.8 mmol/L and CRP < 2 mg/L), and the double high group (RC ≥ 0.8 mmol/L and CRP ≥ 2 mg/L). The association between each group and the risk of MACE was assessed using the multivariate cox proportional hazards regression model, constructed as described above. The cumulative incidence of MACE across the four groups was estimated using the Kaplan–Meier method, and differences between groups were compared using the log-rank test. Multiplicative interaction was assessed by introducing a product term of elevated RC (yes/no) and elevated CRP (yes/no) into the cox regression model. To evaluate whether the combined effect of RC and CRP varied across different populations, we compared the magnitude and statistical significance of effect estimates across subgroups defined by age, sex, smoking, and drinking to assess potential effect modification.

To further explore the impact of the combined RC and CRP grouping on different types of cardiovascular events, the low-low group was set as the reference. Using the multivariate cox proportional hazards regression model, we evaluated the associations between the three exposure groups and the risks of non-fatal MI, non-fatal stroke, and cardiovascular death, respectively, with adjustment for covariates as described below. Given that cardiovascular death may be subject to competing risk from non-cardiovascular death, we further applied the Fine-Gray competing risk model to obtain more robust risk estimates, using non-cardiovascular death as the competing event. Sub-distribution hazard ratios (sHRs) were calculated for cardiovascular death.

### Cholesterol-lowering medication uses on MACE risk associated with the combination of RC and CRP

To investigate the potential effect modification by self-reported cholesterol-lowering medication uses on the association between the combined RC and CRP exposure and the risk of MACE, participants were divided into non-users and users based on baseline self-reported cholesterol-lowering medication use. In each subgroup, the multivariate Cox proportional hazards regression model was used to evaluate the association between the three exposure groups and the risk of MACE, with the low-low group as the reference.

Similarly, to examine the potential interaction between cholesterol-lowering medication uses and RC and CRP, and to formally test whether the observed effect differences in the stratified analysis were statistically significant, we constructed Cox regression models including interaction terms. Specifically, product terms were introduced for cholesterol-lowering medication use (yes/no) with elevated RC (yes/no), and for cholesterol-lowering medication use (yes/no) with elevated CRP (yes/no), to evaluate multiplicative interaction on the risk of MACE. Additionally, to further explore the role of cholesterol-lowering medication uses under different RC backgrounds, we assessed the impact of elevated CRP on MACE risk separately in the RC < 0.8 mmol/L and RC ≥ 0.8 mmol/L populations, thereby examining potential effect modification by cholesterol-lowering medication uses. Finally, to assess the robustness of the findings, cutoffs were redefined based on the median and the 75th percentile of RC and CRP.

### Statistical analysis

Previous studies have established that elevated RC, commonly defined as ≥0.8 mmol/L, is associated with an increased risk of MI and cardiovascular death (19). Additionally, a CRP level ≥2 mg/L is widely used to identify residual inflammatory risk in CVD (21–23). Therefore, in this study, RC was categorized as < 0.8 or ≥0.8 mmol/L, and CRP was categorized as < 2 or ≥2 mg/L. To investigate the association between the combined RC and CRP grouping and the risk of MACE, 376,817 participants were divided into four groups based on the predefined cutoffs: the low-low group (RC < 0.8 mmol/L and CRP < 2 mg/L), the single elevated CRP group (RC < 0.8 mmol/L and CRP ≥ 2 mg/L), the single elevated RC group (RC ≥ 0.8 mmol/L and CRP < 2 mg/L), and the double high group (RC ≥ 0.8 mmol/L and CRP ≥ 2 mg/L). Normality was assessed using the Shapiro–Wilk test, which indicated that most continuous variables were skewed (*P* < 0.05). Accordingly, continuous variables were presented as median (25th percentile, 75th percentile), and categorical variables as frequency (percentage). Differences in continuous variables across the four groups were evaluated using the Jonckheere–Terpstra trend test. For ordered categorical variables (including binary variables), the Cochran–Armitage trend test was applied, while unordered categorical variables were compared using the Pearson chi-square test. Three Cox proportional hazards models were constructed with stepwise adjustment for covariates. Model 1 was adjusted for age, sex, smoke, drink, ethnicity, diet score, and physical activity. Model 2 was further adjusted for LDL-C and TG based on Model 1. Model 3 additionally included diabetes, hypertension, antihypertensive agent use, and hypoglycemic agent use. Missing covariate data were handled using multiple imputation by chained equations. The highest missing rate was observed for physical activity (21.99%), followed by hypertension (7.15%) and diabetes (5.33%), while missing rates were 0.39% for smoking and 0.16% for drinking (Supplementary Table 1).

## Results

### Individual associations of CRP and RC with MACE

A total of 376,817 participants were included in this study. Cox proportional hazards regression analysis revealed that both CRP and RC levels were significantly associated with the risk of MACE (Table 1). For CRP, compared with the < 2 mg/L group as the reference, the risk of MACE in the ≥2 mg/L group was 16.8% higher after full adjustment for covariates (HR = 1.168, 95% CI: 1.129–1.207). Similarly, in the analysis of RC, compared with the < 0.8 mmol/L reference group, the risk of MACE in the ≥0.8 mmol/L group increased by 7.4% (HR = 1.074, 95% CI: 1.024–1.126).

**Table 1:** Cox model assessing the impact of CRP levels and RC levels on MACE risk.

	HR (95% CI)		
	Model 1	Model 2	Model 3
CRP < 2 mg/L	1 (reference)	1 (reference)	1 (reference)
CRP ≥ 2 mg/L	1.233 (1.194–1.274)	1.192 (1.153–1.232)	1.168 (1.129–1.207)
RC < 0.8 mmol/L	1 (reference)	1 (reference)	1 (reference)
RC ≥ 0.8 mmol/L	1.257 (1.217–1.299)	1.070 (1.020–1.123)	1.074 (1.024–1.126)

Values are HR (95% CI) derived from Cox regression models.

Model 1 was adjusted for age, sex, smoke, drink, ethnicity, diet score, physical activity.

Model 2 was adjusted as Model 1 plus LDL-C, TG.

Model 3 was adjusted as Model 2 plus diabetes, hypertension, antihypertensive agent, hypoglycemic agent.

CI, confidence interval; CRP, C-reactive protein; HR, hazard ratio; LDL-C, low density lipoprotein cholesterol; RC, remnant cholesterol; TG, triglycerides.

The Kaplan–Meier survival analysis further confirmed the association over time. The incidence of MACE in the CRP ≥ 2 mg/L group was significantly higher throughout follow-up compared to the CRP < 2 mg/L group, and the difference in cumulative incidence between the two groups gradually widened with increasing follow-up time (log-rank *P* ≤ 0.001, Figure 2A). The cumulative event rate in the RC ≥ 0.8 mmol/L group was also higher than that in the RC < 0.8 mmol/L group (log-rank *P* ≤ 0.001, Figure 2B). Together with the previous results for CRP, these findings demonstrate the association of both high RC levels and high inflammatory levels with adverse cardiovascular prognosis.

**Figure 2:**
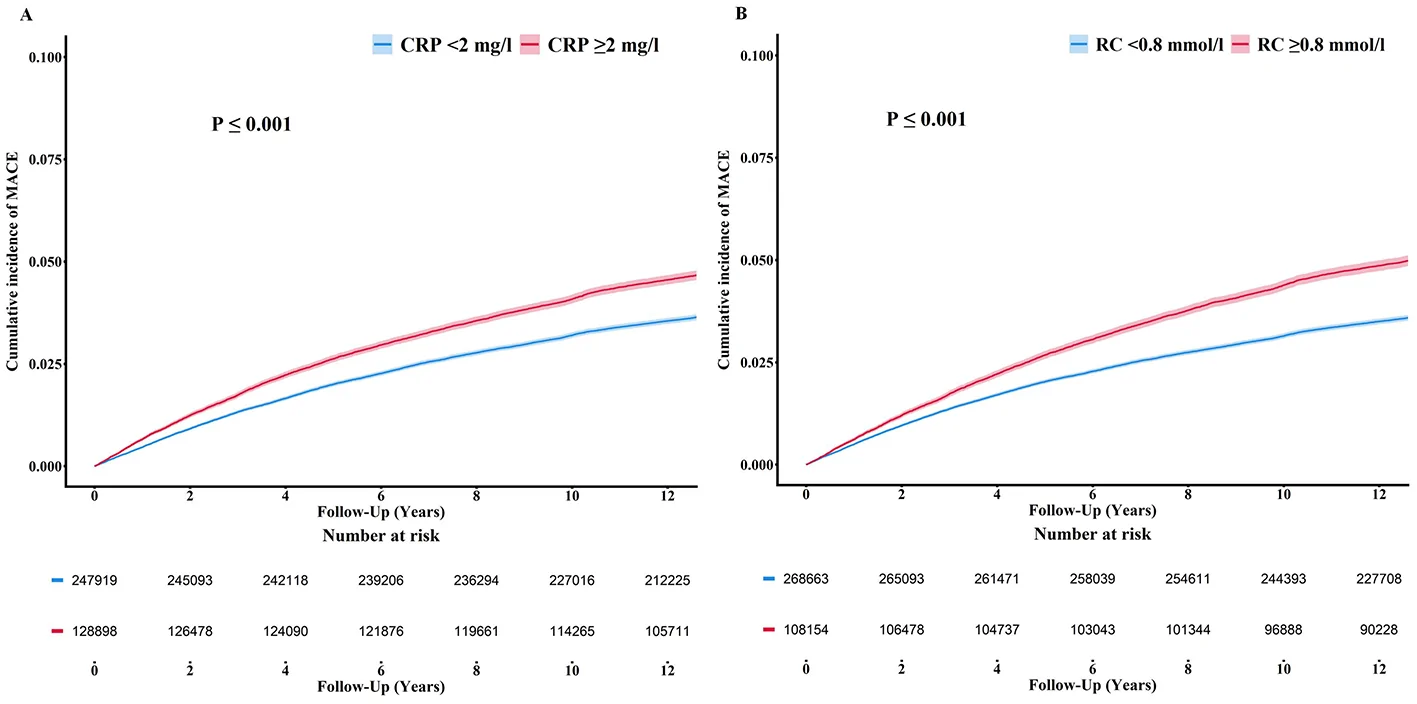
Kaplan–Meier survival curves for MACE according to RC and CRP levels. **(A)** Association between CRP level and MACE risk. The blue curve represents CRP < 2 mg/L, and the solid red curve represents CRP ≥2 mg/L. The difference between the two groups was statistically significant (*P* ≤ 0.001). **(B)** Association between RC level and MACE risk. The blue curve represents RC < 0.8 mmol/L, and the solid red curve represents RC ≥0.8 mmol/L. The difference between the two groups was statistically significant (*P* ≤ 0.001). Numbers at risk are shown below each panel at 0, 2, 4, 6, 8, 10, and 12 years of follow-up. CRP, C-reactive protein; RC, remnant cholesterol.

### Demographic characteristics

The analysis of the combined effects of RC and CRP on MACE comprised a total of 376,817 participants. Based on the imputed complete dataset, we divided all participants into four groups according to RC and CRP levels (cutoffs: 0.8 mmol/L and 2 mg/L, respectively). Compared with the low-RC/low-CRP reference group, the single elevated RC group, the single elevated CRP group, and the double-high group all differed significantly in multiple baseline cardiovascular risk factors (Table 2). There were significant differences in age distribution among the groups. In terms of gender composition, compared with the low-low group, the proportion of males was higher in the single elevated CRP group, while the proportion of females was higher in the single elevated RC group. In addition to the expected differences in the two grouping indicators themselves, several other cardiovascular risk factors showed distinct patterns across the four groups. Participants in the double high group had higher levels of TG and LDL-C compared with the other groups. This group also had the highest prevalence of hypertension and the highest proportion with a smoking history. Regarding physical activity, the double high group had the highest proportion of low-intensity activity. Similarly, dietary health scores showed a stepwise worsening across groups, with the double high group having the highest proportion of unhealthy diets. Furthermore, there were significant differences among groups in diabetes prevalence and the proportion of hypoglycemic agent use. Among them, the single elevated CRP group had higher rates of both compared with the other groups.

**Table 2:** Baseline characteristics of the study participants in the total cohort.

Characteristics	Group 1 (*n* = 186,149)	Group 2 (*n* = 82,514)	Group 3 (*n* = 61,770)	Group 4 (*n* = 46,384)	*P*-trend
Age (years)	56.00 (48.00–62.00)	58.00 (50.00–63.00)	57.00 (50.00–63.00)	58.00 (52.00–63.00)	< 0.001
Gender					< 0.001
Male	108,405 (58.2%)	50,881 (61.7%)	26,523 (42.9%)	25,819 (55.7%)	
Female	77,744 (41.8%)	31,633 (38.3%)	35,247 (57.1%)	20,565 (44.3%)	
Ethnicity					< 0.001
White	168,236 (90.4%)	74,728 (90.6%)	56,749 (91.9%)	42,755 (92.2%)	
Smoking history					< 0.001
Yes	106,668 (57.3%)	49,366 (59.8%)	36,477 (59.1%)	29,037 (62.6%)	
No	79,481 (42.7%)	33,148 (40.2%)	25,293 (40.9%)	17,347 (37.4%)	
Drinking history					< 0.001
Yes	179,119 (96.2%)	78,113 (94.7%)	59,519 (96.4%)	44,069 (95.0%)	
No	7,030 (3.8%)	4,401 (5.3%)	2,251 (3.6%)	2,315 (5.0%)	
Physical activity					< 0.001
Low intensity	28,999 (15.6%)	17,166 (20.8%)	11,101 (18.0%)	10,495 (22.6%)	
Moderate intensity	96,167 (51.7%)	41,191 (49.9%)	31,681 (51.3%)	22,866 (49.3%)	
High intensity	60,983 (32.8%)	24,157 (29.3%)	18,988 (30.7%)	13,023 (28.1%)	
TG (mmol/L)	1.16 (0.88–1.55)	1.36 (1.04–1.78)	2.32 (1.76–3.04)	2.48 (1.93–3.21)	< 0.001
LDL-C (mmol/L)	3.31 (2.84–3.79)	3.32 (2.83–3.80)	4.26 (3.77–4.76)	4.27 (3.78–4.77)	< 0.001
Hypertension	85,140 (45.7%)	48,681 (59.0%)	33,879 (54.8%)	30,052 (64.8%)	< 0.001
Diabetes	10,590 (5.7%)	9,268 (11.2%)	2,818 (4.6%)	4,805 (10.4%)	< 0.001
Antihypertensive agent	7,062 (3.8%)	6,755 (8.2%)	2,655 (4.3%)	4,460 (9.6%)	< 0.001
Hypoglycemic agent	5,385 (2.9%)	3,852 (4.7%)	629 (1.0%)	871 (1.9%)	< 0.001
Diet score					< 0.001
0	1,867 (1.0%)	1,178 (1.4%)	962 (1.6%)	847 (1.8%)	
1	15,898 (8.5%)	8,705 (10.5%)	6,747 (10.9%)	5,776 (12.5%)	
2	41,171 (22.1%)	20,427 (24.8%)	15,158 (24.5%)	12,127 (26.1%)	
3	56,516 (30.4%)	25,032 (30.3%)	18,725 (30.3%)	13,691 (29.5%)	
4	48,801 (26.2%)	19,372 (23.5%)	14,388 (23.3%)	10,212 (22.0%)	
5	21,896 (11.8%)	7,800 (9.5%)	5,790 (9.4%)	3,731 (8.0%)	

The baseline characteristics of the study participants were divided into four groups (Group 1–Group 4). Continuous variables are presented as median (interquartile range), and categorical variables are described as frequency (percentage). The Jonckheere-Terpstra trend test was used for continuous variables; the Cochran–Armitage trend test was used for binary and ordered categorical variables; and Pearson's chi-square test was used for unordered categorical variables.

Physical activity levels were quantified using metabolic equivalent (MET) hours per week and categorized as: low intensity (< 10 MET-hours/week), moderate intensity (10–50 MET-hours/week), and high intensity (>50 MET-hours/week).

Diet score was derived from the following dietary factors: vegetable intake ≥4 tablespoons/day, fruit intake ≥3 pieces/day, fish intake ≥twice a week, unprocessed red meat intake ≤ twice a week, and processed meat intake ≤ twice a week. Each beneficial dietary factor received one point, with the healthy eating score ranging from 0 to 5.

Group 1: RC < 0.8 mmol/L and CRP < 2 mg/L; Group 2: RC < 0.8 mmol/L and CRP ≥ 2 mg/L; Group 3: RC ≥ 0.8 mmol/L and CRP < 2 mg/L; Group 4: RC ≥ 0.8 mmol/L and CRP ≥ 2 mg/L.

LDL-C, low density lipoprotein cholesterol; TG, triglycerides.

To evaluate the influence of cholesterol-lowering medications, the final analytic cohort comprised 373,688 individuals. Baseline characteristics differed significantly across the four groups defined by the RC (0.8 mmol/L) and CRP (2 mg/L) cutoffs. Compared with the reference group the single CRP-elevated group had a higher proportion of males and exhibited the highest prevalence of diabetes and use of glucose-lowering agents, whereas the single RC-elevated group showed a slightly higher female proportion. The dual-high group manifested the most unfavorable cardiovascular risk profile, characterized by older age, the highest smoking rate, less physical activity, the poorest dietary habits, as well as the highest levels of TG, LDL, and the greatest prevalence of hypertension (Supplementary Table 2).

To assess potential bias due to missing data, we compared baseline characteristics between participants included in the final analysis and those excluded because of missing covariate data (Supplementary Table 3). Significant differences were observed for most characteristics, including age, gender, drinking habits, race, dietary score, physical activity status, LDL-C, hypertension, antihypertensive agent use, and hypoglycemic agent use (all *P* < 0.05). Although there were significant differences in some indicators, the absolute differences were relatively small, suggesting that selection bias due to missing data may be limited.

### Association of the combined effect of CRP and RC with MACE risk

Using the low-low group as the reference, we examined the combined impact of RC and CRP on the risk of MACE. After stepwise adjustment for covariates, the other three groups all showed significantly increased risks, with the largest increase observed in the double high group (HR = 1.239, 95% CI: 1.167–1.316), corresponding to a 23.9% higher risk (Table 3). Kaplan–Meier survival analysis further demonstrated a progressively worsening long-term cardiovascular prognosis with the accumulation of risk factors (Figure 3). To further explore the interaction between RC and CRP on the risk of MACE, the multiplicative interaction analysis showed that the HR for the product term of RC and CRP was 1.138 (95% CI: 1.084–1.195), indicating a positive multiplicative interaction between the two factors. Furthermore, stratified analyses by age and sex were performed for the four combined groups (Supplementary Figure 1). In all subgroups with the exception of the non-drinking group, the dual-high group exhibited a significant association with MACE risk (all *P* < 0.001). However, given the relatively small sample size of the non-drinking subgroup, the statistical power was limited.

**Table 3:** Cox model assessing combined impact of CRP and RC levels on MACE risk.

	HR (95% CI)		
RC level and CRP level	Model 1	Model 2	Model 3
RC < 0.8 mmol/L and CRP < 2 mg/L	1 (reference)	1 (reference)	1 (reference)
RC < 0.8 mmol/L and CRP ≥ 2 mg/L	1.235 (1.185–1.287)	1.217 (1.168–1.269)	1.192 (1.143–1.243)
RC ≥ 0.8 mmol/L and CRP < 2 mg/L	1.263 (1.209–1.319)	1.092 (1.034–1.155)	1.095 (1.036–1.158)
RC ≥ 0.8 mmol/L and CRP ≥ 2 mg/L	1.474 (1.407–1.544)	1.254 (1.182–1.331)	1.239 (1.167–1.316)
RC^*^CRP	1.138 (1.084–1.195) *P* < 0.001		

Values are HR (95% CI) derived from Cox regression models.

Model 1 was adjusted for age, sex, smoke, drink, ethnicity, diet score, physical activity.

Model 2 was adjusted as Model 1 plus LDL-C, TG.

Model 3 was adjusted as Model 2 plus diabetes, hypertension, antihypertensive agent, hypoglycemic agent.

CI, confidence interval; CRP, C-reactive protein; HR, hazard ratio; LDL-C, low density lipoprotein cholesterol; RC, remnant cholesterol; TG, triglycerides.

**Figure 3:**
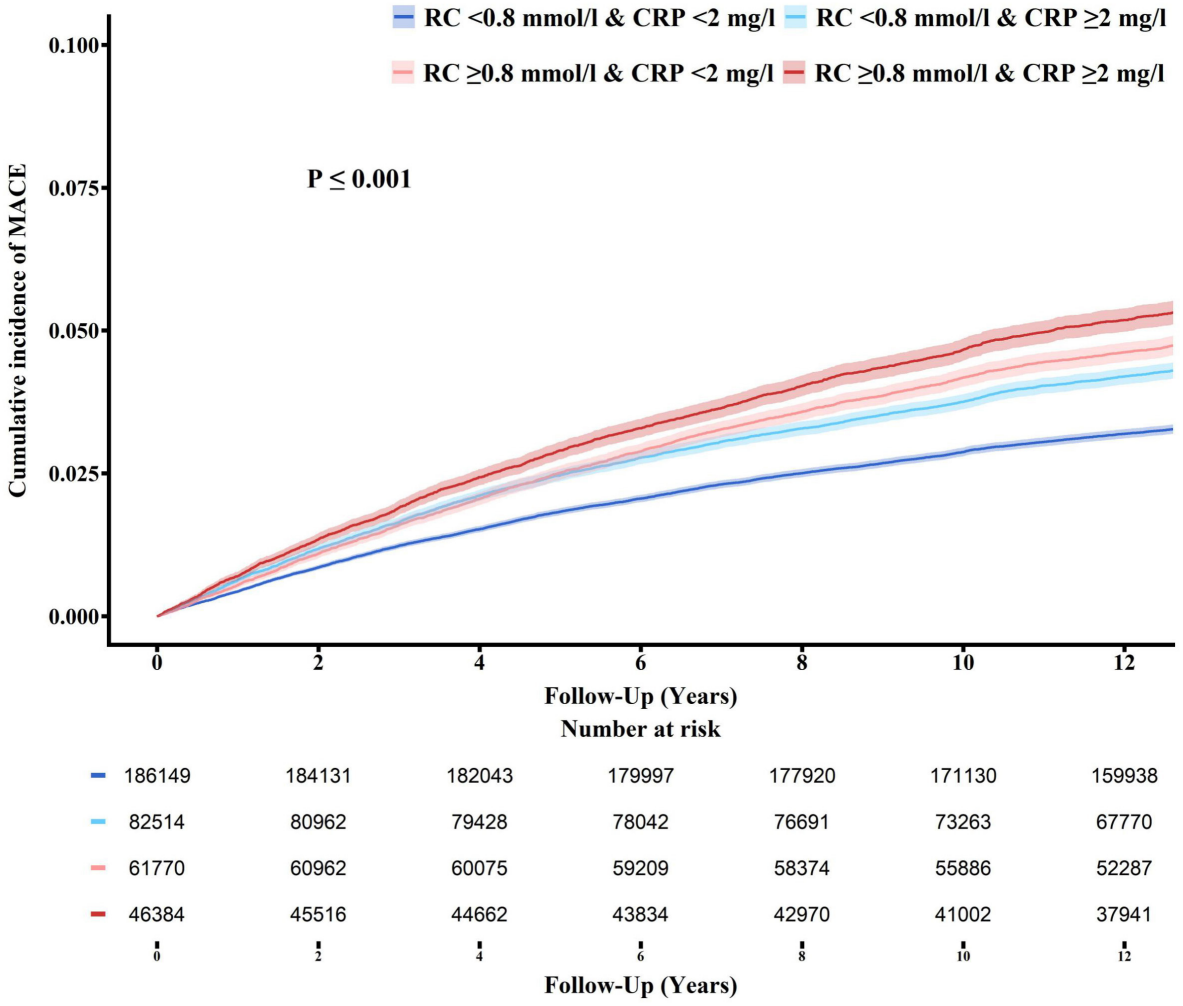
Kaplan–Meier survival curves for MACE according to combined RC and CRP levels. The dark blue line represents patients with RC < 0.8 mmol/L and CRP < 2 mg/L; the light blue line represents RC < 0.8 mmol/L and CRP ≥2 mg/L; the light red line represents RC ≥ 0.8 mmol/L and CRP < 2 mg/L; the dark red line represents RC ≥ 0.8 mmol/L and CRP ≥ 2 mg/L. Numbers at risk are shown below the figure at 0, 2, 4, 6, 8, 10, and 12 years of follow-up. The difference among the four groups was statistically significant (*P* ≤ 0.001). CRP, C-reactive protein; RC, remnant cholesterol.

We further analyzed the impact of the combined RC and CRP grouping on the individual components of MACE, including non-fatal MI, non-fatal stroke, and cardiovascular death (Table 4). The analysis revealed distinct patterns for the individual MACE components. The risk of non-fatal MI was significantly increased in all three exposure groups (all *P* < 0.05). For non-fatal stroke, an increased risk was observed only in the double high group. The risk of cardiovascular death was also increased in both the single elevated CRP group and the double high group (all *P* < 0.05). In the Fine-Gray competing risk model with non-cardiovascular death as the competing event, the risk of cardiovascular death remained significantly higher in the single elevated CRP group and the double high group (*P* < 0.05). Although the association in the single elevated RC group was not statistically significant, the direction of the effect was consistent with that observed in the main analysis (Supplementary Table 4).

**Table 4:** Cox model of combined CRP and RC effect on non-fatal MI, non-fatal stroke, and cardiovascular death.

Non-fatal MI-HR (95% CI)			
RC level and CRP level	Model 1	Model 2	Model 3
RC < 0.8 mmol/L and CRP < 2 mg/L	1 (reference)	1 (reference)	1 (reference)
RC < 0.8 mmol/L and CRP ≥ 2 mg/L	1.126 (1.068–1.187)	1.105 (1.048–1.165)	1.095 (1.037–1.155)
RC ≥ 0.8 mmol/L and CRP < 2 mg/L	1.370 (1.300–1.444)	1.115 (1.042–1.193)	1.116 (1.043–1.195)
RC ≥ 0.8 mmol/L and CRP ≥ 2 mg/L	1.409 (1.328–1.494)	1.122 (1.041–1.209)	1.121 (1.039–1.208)
**Non-fatal stroke-HR (95% CI)**			
RC level and CRP level	Model 1	Model 2	Model 3
RC < 0.8 mmol/L and CRP < 2 mg/L	1 (reference)	1 (reference)	1 (reference)
RC < 0.8 mmol/L and CRP ≥ 2 mg/L	1.113 (1.006–1.232)	1.120 (1.011–1.239)	1.106 (0.999–1.226)
RC ≥ 0.8 mmol/L and CRP < 2 mg/L	1.109 (0.994–1.237)	1.033 (0.900–1.185)	1.028 (0.895–1.179)
RC ≥ 0.8 mmol/L and CRP ≥ 2 mg/L	1.267 (1.127–1.425)	1.185 (1.023–1.374)	1.174 (1.012–1.361)
**Cardiovascular death-HR (95% CI)**			
RC level and CRP level	Model 1	Model 2	Model 3
RC < 0.8 mmol/L and CRP < 2 mg/L	1 (reference)	1 (reference)	1 (reference)
RC < 0.8 mmol/L and CRP ≥ 2 mg/L	1.149 (1.057–1.248)	1.143 (1.052–1.242)	1.137 (1.046–1.236)
RC ≥ 0.8 mmol/L and CRP < 2 mg/L	1.128 (1.017–1.252)	1.082 (0.953–1.228)	1.089 (0.960–1.236)
RC ≥ 0.8 mmol/L and CRP ≥ 2 mg/L	1.302 (1.183–1.432)	1.240 (1.094–1.404)	1.244 (1.098–1.410)

Values are HR (95% CI) derived from Cox regression models.

Model 1 was adjusted for age, sex, smoke, drink, ethnicity, diet score, physical activity.

Model 2 was adjusted as Model 1 plus LDL-C TG.

Model 3 was adjusted as Model 2 plus diabetes, hypertension, antihypertensive agent, hypoglycemic agent.

CI, confidence interval; CRP, C-reactive protein; HR, hazard ratio; LDL-C, low density lipoprotein cholesterol; MI, myocardial infarction; RC, remnant cholesterol; TG, triglycerides.

### The impact of cholesterol-lowering medication uses on the CRP-RC combination-associated risk of MACE

Further stratified analysis was conducted based on cholesterol-lowering medication uses use to explore its effect modification on the association between the combined grouping and the risk of MACE (Supplementary Table 5). In the subgroup not taking cholesterol-lowering medication uses, the risks of MACE in the single elevated CRP group, the single elevated RC group, and the double high group were all increased (all *P* < 0.05). In the subgroup of participants receiving cholesterol-lowering medication uses, the association for the single elevated RC group ceased to be significant, whereas that for the single elevated CRP group and the dual-high group persisted, indicating effect modification by cholesterol-lowering medication on the risk associated with single RC elevation, and suggesting that the association was attenuated among medication users. Although cholesterol-lowering medication use was associated with MACE risk in individuals with elevated RC, the association between CRP elevation and MACE risk appeared similar regardless of RC status, suggesting that additional anti-inflammatory measures may still be warranted (Supplementary Table 6). Multiplicative interaction tests (Supplementary Table 7) demonstrated a significant interaction between cholesterol-lowering medication uses and RC (HR = 0.606, 95% CI: 0.526–0.698, *P* < 0.001), whereas the interaction term for cholesterol-lowering medication uses and CRP did not achieve statistical significance (*P* = 0.582). To further evaluate the robustness of the grouping approach, we employed the median (RC ≥ 0.645 mmol/L, CRP ≥ 1.29 mg/L) and the 75th percentile (RC ≥ 0.834 mmol/L, CRP ≥ 2.67 mg/L) as alternative cutoffs, reassessed the joint effect of RC and CRP on MACE risk, and conducted stratified analyses by cholesterol-lowering medication use (Supplementary Tables 8 and 9). The findings were consistent with the primary results. Collectively, these results provide evidence of effect modification by cholesterol-lowering medication use on RC-related cardiovascular risk, while also suggesting that for the cardiovascular risk associated with concurrent elevations in both RC and CRP, supplementary anti-inflammatory interventions may be warranted.

## Discussion

Based on a large prospective cohort of 376,817 participants with a median follow-up of 13.4 years, this study systematically investigated the individual and joint associations of RC and CRP with the risk of MACE. The results demonstrated that both RC and CRP were independent risk factors for MACE. Compared with either marker alone, the combined elevation of RC and CRP conferred the highest risk of MACE, corresponding to a 23.9% increase. Multiplicative interaction analysis further revealed a significant positive interaction between RC and CRP on MACE risk. Furthermore, self-reported cholesterol-lowering medication use exhibited a differential modifying effect across risk strata: it was associated with attenuation of cardiovascular risk in individuals with isolated RC elevation, but not in those with isolated CRP elevation or concurrent elevation of both markers. These findings suggest that lipid-driven and inflammation-driven residual risks may require distinct management strategies.

Atherosclerosis is a complex pathological process involving lipid deposition and inflammatory responses (24, 25). RC particles can penetrate the vascular endothelium and induce local oxidative stress and inflammatory reactions (26, 27). In this study, multiplicative interaction analysis revealed a significant positive interaction between RC and CRP on MACE risk, indicating that the combined effect of concurrent RC and CRP elevation was greater than the product of their individual effects on the multiplicative scale. This multiplicative pattern may reflect synergistic amplification between lipid-driven and inflammatory pathways, although the underlying mechanisms require further investigation with longitudinal biomarker data. Importantly, the double-high group exhibited the highest absolute MACE risk (HR = 1.239), underscoring the clinical importance of combined risk stratification. Recent studies have proposed composite indices combining RC and CRP (e.g., RCII) and demonstrated their associations with stroke, cardiovascular mortality, and coronary heart disease (28–30), but none formally evaluated the multiplicative interaction between the two factors. This study extends the literature by systematically demonstrating a significant super-multiplicative interaction between RC and CRP in a large general population.

Additionally, this study analyzed the impact of combined RC and CRP grouping on individual MACE components. The risk patterns associated with combined exposure differed across cardiovascular endpoints. The association with non-fatal MI was the most robust, with significant risk increases observed in the isolated CRP elevation, isolated RC elevation, and double-high groups. This finding is consistent with previous studies (31–33), while further validating the incremental value of combined assessment for this endpoint. In contrast, non-fatal stroke risk was significantly increased only in the double-high group. This differs from previous reports where isolated elevations of either marker were associated with stroke risk (11, 34, 35). This discrepancy may relate to the heterogeneity of stroke etiology. Unlike MI which is predominantly driven by coronary atherosclerosis, stroke encompasses multiple subtypes, including large artery atherosclerosis, cardio-embolism, and small vessel occlusion, with potentially differential sensitivity to lipid metabolic disturbances and inflammation (36, 37). Our findings suggest that combined assessment of RC and CRP may offer greater value for stroke risk evaluation, though this warrants further validation. Regarding cardiovascular death, both the isolated CRP elevation and double-high groups showed significantly increased risk, whereas the isolated RC elevation group did not reach statistical significance in competing risk models. This aligns with evidence that inflammation is more directly associated with mortality, and that the effect of RC may be partially mediated through inflammatory pathways. These findings indicate that the impact of combined RC and CRP exposure varies across cardiovascular endpoints, highlighting the value of detailed component analyses for understanding their complex roles in CVD and informing individualized risk assessment.

We further explored the impact of self-reported cholesterol-lowering medication use on RC- and CRP-related risks through stratified analysis. These medications were associated with attenuation of MACE risk in the isolated RC elevation group, but not in the isolated CRP elevation or double-high groups. This differential association is mechanistically plausible: cholesterol-lowering medications primarily reduce cholesterol synthesis and lower atherogenic lipoproteins including RC (38), and would therefore be expected to attenuate risk when RC is elevated. In contrast, risk in isolated CRP elevation is predominantly driven by non-lipid-dependent inflammatory pathways, against which the pleiotropic anti-inflammatory effects of these medications appear insufficient (39). In the double-high group, lowering RC alone did not eliminate excess risk, suggesting that persistent CRP elevation may maintain residual inflammatory risk even when RC is partially reduced. These findings indicate that for inflammation-driven residual risk, particularly when both RC and CRP are elevated, anti-inflammatory strategies beyond cholesterol-lowering medications (e.g., targeting IL-1β or IL-6 pathways) may be warranted. However, translation of these strategies into clinical practice faces several barriers: clinical benefit evidence remains limited and inconsistent, colchicine shows efficacy in chronic coronary syndromes but not definitively in acute settings (40), and the recent phase 3 Ziltivekimab Cardiovascular Outcomes Trial (ZEUS) found that the anti-IL-6 antibody ziltivekimab, despite reducing hsCRP, failed to reduce MACE (41, 42); safety concerns persist, as anti-IL-1β monoclonal antibodies increase fatal infections (43) and colchicine causes gastrointestinal side effects (40); optimal patient selection requires biomarker guidance (e.g., hsCRP), which is not yet standardized; and cost and accessibility issues remain, with canakinumab costly and unapproved for cardiovascular use, while colchicine suffers from low physician awareness despite FDA approval since 2023.

This study also has several important limitations. First, this is an observational epidemiological analysis based on a large biobank, and the study design itself precludes causal inference. Although multivariable adjustment was performed in the statistical analyses, the possibility of residual confounding by unmeasured factors (e.g., prior revascularization history and other subclinical inflammatory diseases) cannot be completely excluded.

Second, participants in the UKB are subject to a “healthy volunteer” bias, and a considerable number of individuals were excluded from this analysis due to missing data on key variables such as RC and CRP. These excluded individuals tend to have higher cardiovascular risk profiles, which may affect the generalizability of the findings and introduce unpredictable bias into the observed risk associations. Furthermore, the sample size of the non-drinking subgroup in the present study was relatively limited, which may have resulted in insufficient statistical power, particularly in stratified analyses aimed at detecting potential interaction effects or intergroup differences; accordingly, any inferences drawn from this subgroup should be interpreted with caution.

Third, information on cholesterol-lowering medication use was obtained only from self-report at the baseline visit, with no data on dosage, frequency, long-term adherence, or changes in medication use during follow-up. This time-invariant crude classification not only hinders the exploration of dose-response relationships but also introduces misclassification bias, which may substantially underestimate or obscure the true effects of cholesterol-lowering medication uses. Furthermore, the present study dichotomized cholesterol-lowering medication as a binary variable (yes/no) without distinguishing between different drug classes (e.g., statins, ezetimibe, PCSK9 inhibitors). The mechanisms of action, cholesterol-lowering potency, and effects on RC and inflammatory markers vary considerably across drug classes, and this crude categorization may further mask class-specific effect modifications. Therefore, all conclusions related to cholesterol-lowering medication should be considered hypothesis-generating rather than confirmatory. Future studies incorporating repeated measurements and detailed information on drug classes are needed to clarify the dynamic relationships between these factors and cardiovascular risk.

Fourth, key biomarkers (RC and CRP) were assessed only once at enrolment and could not capture changes during follow-up. In this study, non-fasting serum samples were used, and LDL-C was measured by the enzyme protection method. Even after excluding individuals with TG ≥ 5.7 mmol/L, the enzyme protection method may still be subject to interference from lipemia and VLDL particles when TG levels are in the moderate range, leading to certain inaccuracies in LDL-C measurements. RC was calculated using the formula RC = TC–HDL–C–LDL–C; therefore, any measurement error in LDL-C is directly propagated to RC, affecting its accuracy. In addition, CRP was measured by latex-enhanced immunoturbidimetry using high-sensitivity reagents. Although this method has high sensitivity, a single measurement cannot capture the long-term inflammatory exposure profile. As static indicators, single measurements of biomarkers are unlikely to reflect dynamic changes during follow-up or their long-term relationships with cardiovascular risk. Future studies should employ repeated measurements or more accurate direct methods (e.g., ultracentrifugation or β-quantification) to further elucidate the joint risk effects of RC and CRP.

Fifth, over 90% of UKB participants are of White European ancestry, which limits the generalizability of the findings to other racial/ethnic groups or populations in different regions. The ascertainment of the primary endpoint (MACE) and other diseases relied on ICD-10 coding, which may introduce misclassification. In addition, this study focused on long-term risk in the general population and lacked data on perioperative patients, surgery-related outcomes, or perioperative management; therefore, the results cannot be directly applied to clinical decision-making in the surgical setting.

## Conclusion

This large-scale prospective study demonstrates that concurrent elevation of RC and CRP is associated with the highest risk of MACE, with a synergistic effect exceeding the sum of their individual contributions. Notably, self-reported cholesterol-lowering medication use was associated with risk reduction in individuals with isolated RC elevation, but not in those with concurrent RC and CRP elevation; isolated inflammatory risk may require additional anti-inflammatory strategies. Combined evaluation of RC and CRP may facilitate more personalized cardiovascular prevention.

## Data Availability

The original contributions presented in the study are included in the article/Supplementary material, further inquiries can be directed to the corresponding authors.
