# Supplementary material for: Remnant cholesterol, C-reactive protein, cholesterol-lowering medication, and cardiovascular outcome: novel findings from UK Biobank
Source: Front Nutr. 2026 Oct 5;13:1920077. doi: 10.3389/fnut.2026.1920077 (PMC13677500; doi:10.3389/fnut.2026.1920077)
Supplement: Supplementary file 1 [file Table_1.docx]

**Remnant cholesterol, C-reactive protein, cholesterol-lowering medication, and cardiovascular outcome: novel findings from UK Biobank**

**Supplementary Figure**

**Supplementary Figure 1. Subgroup analysis of the cox proportional hazards model for combined RC and CRP on MACE.**

The reference group was defined as RC <0.8 mmol/L and CRP <2 mg/L. The other three groups were RC <0.8 mmol/L and CRP ≥2 mg/L, RC ≥0.8 mmol/L and CRP <2 mg/L, and RC ≥0.8 mmol/L and CRP ≥2 mg/L. All estimates were derived from multivariable Cox proportional hazards models adjusted for age, sex, smoke, drink, ethnicity, diet score, physical activity, LDL-C, TG, diabetes, hypertension, antihypertensive agent, hypoglycemic agent. The red dashed line indicates HR = 1. P values represent the significance compared with the reference group; P ≤0.05 was considered statistically significant. Subgroup analyses were stratified by sex (male, female), age (<57 years, ≥57 years), smoking status (non-smoker, current smoker), and drinking status (non-drinker, current drinker). Abbreviations: CI, confidence interval; CRP, C-reactive protein; HR, hazard ratio; LDL-C, low density lipoprotein cholesterol; RC, remnant cholesterol; TG, triglycerides.

**Supplementary Table 1. Patterns and proportions of missing data for covariates in the analytical cohort.**

| Rank | Variable | Missing, % |
| --- | --- | --- |
| 1 | Physical activity | 21.99 |
| 2 | Hypertension | 7.15 |
| 3 | Diabetes | 5.33 |
| 4 | Ethnicity | 0.39 |
| 5 | Smoking | 0.39 |
| 6 | Drinking | 0.16 |
| 7 | TG | 0.05 |
| 8-13 | Other covariates | 0.00 |

Notes: N = [376,817]. Other covariates with no missing data include age, sex, LDL-C, diet score, antihypertensive agent and hypoglycemic agent.

Abbreviations: LDL-C, low density lipoprotein cholesterol; TG, triglycerides;

**Supplementary Table 2. Baseline characteristics of the study participants in the restricted cohort**

| Characteristics | Group1  (n = 184,782) | Group2  (n = 81,719) | Group3  (n = 61,254) | Group4  (n = 45,933) | P-trend |
| --- | --- | --- | --- | --- | --- |
| Age (years) | 56.00  (48.00-62.00) | 58.00  (50.00-63.00) | 57.00  (50.00-63.00) | 58.00  (52.00-63.00) | <0.001 |
| Gender |  |  |  |  | <0.001 |
| Male | 107,804 (58.3%) | 50,474 (61.8%) | 26,370 (43.1%) | 25,642 (55.8%) |  |
| Female | 76,978 (41.7%) | 31,245 (38.2%) | 34,884 (56.9%) | 20,291 (44.2%) |  |
| Ethnicity |  |  |  |  | <0.001 |
| White | 167,162 (90.5%) | 74,107 (90.7%) | 56,328 (92.0%) | 42,385 (92.3%) |  |
| Smoking history |  |  |  |  | <0.001 |
| Yes | 105,916 (57.3%) | 48,899 (59.8%) | 36,183 (59.1%) | 28,757 (62.6%) |  |
| No | 78,866 (42.7%) | 32,820 (40.2%) | 25,071 (40.9%) | 17,176 (37.4%) |  |
| Drinking history |  |  |  |  | <0.001 |
| Yes | 177,907 (96.3%) | 77,453 (94.8%) | 59,056 (96.4%) | 43,681 (95.1%) |  |
| No | 6,875 (3.7%) | 4,266 (5.2%) | 2,198 (3.6%) | 2,252 (4.9%) |  |
| Physical activity |  |  |  |  | <0.001 |
| low intensity | 28,734 (15.6%) | 16,998 (20.8%) | 11,003 (18.0%) | 10,383 (22.6%) |  |
| moderate intensity | 95,478 (51.7%) | 40,827 (50.0%) | 31,430 (51.3%) | 22,653 (49.3%) |  |
| high intensity | 60,570 (32.8%) | 23,894 (29.2%) | 18,821 (30.7%) | 12,897 (28.1%) |  |
| TG (mmol/L) | 1.16 (0.88-1.55) | 1.36 (1.04-1.78) | 2.31 (1.76-3.04) | 2.48 (1.93-3.21) | <0.001 |
| LDL-C (mmol/L) | 3.31 (2.84-3.79) | 3.32 (2.83-3.80) | 4.26 (3.77-4.76) | 4.27 (3.78-4.77) | <0.001 |
| **Hypertension** | 84,508 (45.7%) | 48,199 (59.0%) | 33,592 (54.8%) | 29,758 (64.8%) | <0.001 |
| **Diabetes** | 10,422 (5.6%) | 9,119 (11.2%) | 2,774 (4.5%) | 4,710 (10.3%) | <0.001 |
| **Antihypertensive agent** | 7,038 (3.8%) | 6,730 (8.2%) | 2,648 (4.3%) | 4,447 (9.7%) | <0.001 |
| **Hypoglycemic agent** | 5,313 (2.9%) | 3,799 (4.6%) | 623 (1.0%) | 854 (1.9%) | <0.001 |
| Diet score |  |  |  |  | <0.001 |
| 0 | 1,845 (1.0%) | 1,166 (1.4%) | 953 (1.6%) | 835 (1.8%) |  |
| 1 | 15,528 (8.4%) | 8,465 (10.4%) | 6,621 (10.8%) | 5660 (12.3%) |  |
| 2 | 40,887 (22.1%) | 20,232 (24.8%) | 15,029 (24.5%) | 12,013 (26.2%) |  |
| 3 | 56,195 (30.4%) | 24,839 (30.4%) | 18,600 (30.4%) | 13,572 (29.5%) |  |
| 4 | 48,545 (26.3%) | 19,261 (23.6%) | 14,291 (23.3%) | 10,146 (22.1%) |  |
| 5 | 21,782 (11.8%) | 7,756 (9.5%) | 5,760 (9.4%) | 3,707 (8.1%) |  |

The baseline characteristics of the study participants were divided into four groups (Group1-Group4). Continuous variables are presented as median (interquartile range), and categorical variables are described as frequency (percentage). The Jonckheere-Terpstra trend test was used for continuous variables; the Cochran–Armitage trend test was used for binary and ordered categorical variables; and Pearson’s chi-square test was used for unordered categorical variables.

Physical activity levels were quantified using metabolic equivalent (MET) hours per week and categorized as: low intensity (< 10 MET-hours/week), moderate intensity (10-50 MET-hours/week), and high intensity (> 50 MET-hours/week).

Diet score was derived from the following dietary factors: vegetable intake ≥4 tablespoons/day, fruit intake ≥ 3 pieces/day, ﬁsh intake ≥twice a week, unprocessed red meat intake ≤twice a week, and processed meat intake ≤twice a week. Each beneficial dietary factor received one point, with the healthy eating score ranging from 0 to 5.

Group 1: RC <0.8 mmol/l & CRP <2 mg/l; Group 2: RC <0.8 mmol/l & CRP ≥2 mg/l; Group 3: RC ≥0.8 mmol/l & CRP <2 mg/l; Group 4: RC ≥0.8 mmol/l & CRP ≥2 mg/l.

Abbreviations: LDL-C, low density lipoprotein cholesterol; TG, triglycerides.

**Supplementary Table 3. Comparison of baseline characteristics between the study participants with and without missing key variables.**

| Characteristics | Overall population  (n = 376,817) | Missing data population  (n = 68,742) | P-value |
| --- | --- | --- | --- |
| Age (years) | 57.00 (49.00-63.00) | 57.00 (49.00-62.00) | <0.001 |
| Gender |  |  | <0.001 |
| Male | 211,628 (56.2%) | 40,131 (58.4%) |  |
| Female | 165,189 (43.8%) | 28,611 (41.6%) |  |
| Ethnicity |  |  | <0.001 |
| White | 340,923 (90.9%) | 61,488 (90.3%) |  |
| Smoking history |  |  | 0.418 |
| Yes | 220,570 (58.8%) | 39,938 (58.6%) |  |
| No | 154,498 (41.2%) | 28,167 (41.4%) |  |
| Drinking history |  |  | <0.001 |
| Yes | 359,981 (95.8%) | 65,129 (95.4%) |  |
| No | 15,950 (4.2%) | 3,125 (4.6%) |  |
| Physical activity |  |  | <0.001 |
| low intensity | 52,615 (17.9%) | 9,344 (18.5%) |  |
| moderate intensity | 149,611 (51.0%) | 25,816 (51.0%) |  |
| high intensity | 91,298 (31.1%) | 15,479 (30.5%) |  |
| TG (mmol/L) | 1.53 (1.06-2.36) | 1.45 (1.03-2.09) | <0.001 |
| LDL-C (mmol/L) | 3.56 (3.01-4.14) | 3.53 (2.98-4.13) | <0.001 |
| Hypertension | 183,980 (48.8%) | 33,120 (48.2%) | 0.002 |
| Diabetes | 25,962 (7.3%) | 5,399 (9.4%) | <0.001 |
| Antihypertensive agent | 31,910 (8.5%) | 5,259 (7.7%) | <0.001 |
| Hypoglycemic agent | 10,737 (2.8%) | 2,116 (3.1%) | <0.001 |
| Diet score |  |  | 0.030 |
| 0 | 4,854 (1.3%) | 882 (1.3%) |  |
| 1 | 37,126 (9.9%) | 7,027 (10.2%) |  |
| 2 | 88,883 (23.6%) | 16,004 (23.1%) |  |
| 3 | 113,964 (30.2%) | 20,884 (30.4%) |  |
| 4 | 92,773 (24.6%) | 16,910 (24.6%) |  |
| 5 | 39,217 (10.4%) | 7,035 (10.2%) |  |

Continuous variables with non-normal distribution are presented as median (interquartile range, IQR); categorical variables are presented as frequency (percentage). Between group comparisons were performed using the Mann Whitney U test for continuous variables and the chi square test for categorical variables.

Missing data population excluded individuals with missing data for any covariate. The covariates included age, sex, smoke, drink, ethnicity, diet score, physical activity, LDL-C, TG, diabetes, hypertension, antihypertensive agent, hypoglycemic agent.

Physical activity levels were quantified using metabolic equivalent (MET) hours per week and categorized as: low intensity (< 10 MET-hours/week), moderate intensity (10-50 MET-hours/week), and high intensity (> 50 MET-hours/week).

Diet score was derived from the following dietary factors: vegetable intake ≥4 tablespoons/day, fruit intake ≥3pieces/day, ﬁsh intake ≥twice a week, unprocessed red meat intake ≤twice a week, and processed meat intake ≤twice a week. Each benefical dietary factor received one point, with the healthy eating score ranging from 0 to 5.

Data on physical activity, diabetes, smoking history, drinking history, ethnicity and antihypertensive agent were missing in 18,103 (26.3%), 11,499 (16.7%), 637 (0.9%), 488 (0.7%), 663 (1.0%) and 663 (1.0%) participants in the missing group, and 83,293 (22.1%), 20,397 (5.4%), 1,749 (0.5%), 886 (0.2%), 1,723 (0.5%) and 1,723 (0.5%) in the complete group, respectively. Percentages in the table are calculated based on non-missing values for each variable.

Abbreviations: LDL-C, low density lipoprotein cholesterol; TG, triglycerides

**Supplementary Table 4. Sub-distribution hazard ratios for cardiovascular death by combined CRP and RC categories in competing risk analysis.**

|  | Cardiovascular death |
| --- | --- |
|  | sHR (95% CI) |
| RC <0.8 mmol/l & CRP <2 mg/l | 1 (reference) |
| RC <0.8 mmol/l & CRP ≥2 mg/l | 1.130 (1.025-1.245) |
| RC ≥0.8 mmol/l & CRP <2 mg/l | 1.105 (0.957-1.279) |
| RC ≥0.8 mmol/l & CRP ≥2 mg/l | 1.230 (1.064-1.421) |

Fine-Gray competing risk models were used to estimate sHR and 95% CI for cardiovascular death, with non-cardiovascular death as the competing event. All models were adjusted for age, sex, smoke, drink, ethnicity, diet score, physical activity, LDL-C, TG, diabetes, hypertension, antihypertensive agent, hypoglycemic agent.

Abbreviations: CI, confidence interval; CRP, C-reactive protein; LDL-C, low density lipoprotein cholesterol; RC, remnant cholesterol; sHR, sub-distribution hazard ratios; TG, triglycerides.

|  | No cholesterol-lowering medication  (N = 324,233) | Cholesterol-lowering medication  (N = 49,455) |
| --- | --- | --- |
|  | HR (95% CI) | HR (95% CI) |
| RC <0.8 mmol/l & CRP <2 mg/l | 1 (reference) | 1 (reference) |
| RC <0.8 mmol/l & CRP ≥2 mg/l | 1.225 (1.164-1.290) | 1.159 (1.078-1.246) |
| RC ≥0.8 mmol/l & CRP <2 mg/l | 1.095 (1.029-1.165) | 1.072 (0.941-1.221) |
| RC ≥0.8 mmol/l & CRP ≥2 mg/l | 1.242 (1.161-1.328) | 1.252 (1.090-1.438) |

**Supplementary Table 5. Cox model assessing combined CRP and RC effect on MACE risk by cholesterol-lowering medication use.**

Values are HR (95% CI) derived from Cox regression models.

Model was adjusted for age, sex, smoke, drink, ethnicity, diet score, physical activity, LDL-C, TG, **diabetes, hypertension, antihypertensive agent, hypoglycemic agent.**

Abbreviations: CI, confidence interval; HR, hazard ratio; CRP, C-reactive protein; LDL-C, low density lipoprotein cholesterol; RC, remnant cholesterol; TG, triglycerides.

**Supplementary Table 6.** **CRP-associated MACE risk stratified by RC and medication in entire cohort.**

|  | No cholesterol-lowering medication  (N = 324,233) | Cholesterol-lowering medication  (N = 49,455) |
| --- | --- | --- |
| **Entire Cohort** | HR (95% CI) | HR (95% CI) |
| CRP <2 mg/l | 1 (reference) | 1 (reference) |
| CRP ≥2 mg/l | 1.186 (1.140-1.233) | 1.161 (1.088-1.238) |
| RC <0.8 mmol/l | 1 (reference) | 1 (reference) |
| RC ≥0.8 mmol/l | 1.066 (1.010-1.125) | 1.077 (0.965-1.201) |
| **With low RC** |  |  |
| CRP <2 mg/l | 1 (reference) | 1 (reference) |
| CRP ≥2 mg/l | 1.204 (1.143-1.269) | 1.159 (1.077-1.246) |
| **With elevated RC** |  |  |
| CRP <2 mg/l | 1 (reference) | 1 (reference) |
| CRP ≥2 mg/l | 1.160 (1.094-1.231) | 1.167 (1.010-1.349) |

Values are HR (95% CI) derived from Cox regression models.

Model was adjusted for age, sex, smoke, drink, ethnicity, diet score, physical activity, LDL-C, TG, diabetes, hypertension, antihypertensive agent, hypoglycemic agent.

Low RC indicates RC <0.8 mmol/l, and elevated RC indicates RC ≥0.8 mmol/l.

Abbreviations: CI, confidence interval; HR, hazard ratio; CRP, C-reactive protein; LDL-C, low density lipoprotein cholesterol; RC, remnant cholesterol; TG, triglycerides.

**Supplementary Table 7. Hazard ratios for multiplicative interaction between cholesterol-lowering medication** **use, CRP, and RC on MACE.**

|  | Unadjusted model | | Fully adjusted model | | |
| --- | --- | --- | --- | --- | --- |
|  | HR (95% CI) | P-value | | HR (95% CI) | P-value |
| Cholesterol-lowering medication* RC | 0.387  (0.338-0.444) | <0.001 | | 0.606  (0.526-0.698) | <0.001 |
| Cholesterol-lowering medication* CRP | 0.995  (0.988-1.002) | 0.163 | | 1.002  (0.995-1.009) | 0.582 |

Values are HR (95% CI) derived from Cox regression models.

Model was adjusted for age, sex, smoke, drink, ethnicity, diet score, physical activity, LDL-C, TG, diabetes, hypertension, antihypertensive agent, hypoglycemic agent.

Abbreviations: CI, confidence interval; HR, hazard ratio; CRP, C-reactive protein; LDL-C, low density lipoprotein cholesterol; RC, remnant cholesterol; TG, triglycerides.

**Supplementary Table 8. Association of CRP and RC (categorized by the median) with MACE in Cox model stratified by medication use.**

|  | Overall population  (N = 373,688) | No cholesterol-lowering medication  (N = 324,233) | Cholesterol-lowering medication  (N = 49,455) |
| --- | --- | --- | --- |
|  | HR (95% CI) | HR (95% CI) | HR (95% CI) |
| RC <0.645 mmol/l & CRP <1.29 mg/l | 1 (reference) | 1 (reference) | 1 (reference) |
| RC <0.645 mmol/l & CRP ≥1.29 mg/l | 1.232 (1.173-1.295) | 1.269 (1.191-1.352) | 1.172 (1.082-1.268) |
| RC ≥0.645 mmol/l & CRP <1.29 mg/l | 1.046 (0.987-1.109) | 1.081 (1.010-1.157) | 1.054 (0.934-1.190) |
| RC ≥0.645 mmol/l & CRP ≥1.29 mg/l | 1.214 (1.148-1.283) | 1.286 (1.204-1.373) | 1.133 (1.012-1.268) |

Values are HR (95% CI) derived from Cox regression models

Model was adjusted for age, sex, smoke, drink, ethnicity, diet score, physical activity, LDL-C, TG, diabetes, hypertension, antihypertensive agent, hypoglycemic agent.

Abbreviations: CI, confidence interval; HR, hazard ratio; CRP, C-reactive protein; LDL-C, low density lipoprotein cholesterol; RC, remnant cholesterol; TG, triglycerides.

**Supplementary Table 9. Association of CRP and RC (categorized by the 75th percentile) with MACE in Cox model stratified by medication use.**

|  | Overall population  (N = 373,688) | No cholesterol-lowering medication  (N = 324,233) | Cholesterol-lowering medication  (N = 49,455) |
| --- | --- | --- | --- |
|  | HR (95% CI) | HR (95% CI) | HR (95% CI) |
| RC <0.834 mmol/l & CRP <2.67 mg/l | 1 (reference) | 1 (reference) | 1 (reference) |
| RC <0.834 mmol/l & CRP ≥2.67 mg/l | 1.234 (1.177-1.285) | 1.259 (1.194-1.328) | 1.219 (1.129-1.316) |
| RC ≥0.834 mmol/l & CRP <2.67 mg/l | 1.099 (1.041-1.161) | 1.089 (1.025-1.158) | 1.090 (0.958-1.240) |
| RC ≥0.834 mmol/l & CRP ≥2.67 mg/l | 1.272 (1.192-1.359) | 1.277 (1.187-1.373) | 1.227 (1.046-1.440) |

Values are HR (95% CI) derived from Cox regression models

Model was adjusted for age, sex, smoke, drink, ethnicity, diet score, physical activity, LDL-C, TG, diabetes, hypertension, antihypertensive agent, hypoglycemic agent.

Abbreviations: CI, confidence interval; HR, hazard ratio; CRP, C-reactive protein; LDL-C, low density lipoprotein cholesterol; RC, remnant cholesterol; TG, triglycerides.

**Supplementary Table 10. UK Biobank Field Codes used to ascertain main diseases.**

Non-Fatal MI defined as the following ICD-10 Codes

I20.x,I21.x,I22.x,I23.x,I24.x,125.x

Non-Fatal Stroke as the following ICD-10 Codes

I60.x,I61.x,I63.x,I64

Any Cardiovascular death defined as the following ICD-10 codes

I20.X, I21.X, I22.X, I23.X, I24.X, I25.X, in addition to death due to any of the codes listed for non-Fatal MI or stroke.

Diabetes as the following ICD-10 Codes

E10.x,E11.x,E12.x,E13.x,E14.x

**Hypertension** as the following ICD-10 Codes

I10,I11.x,I12.x,I13.x,I14.x,I15.x

**Supplementary Table 11.** **UK Biobank medication codes used to define hypoglycemic agent.**

| UKB Code | UKB Drug Category |
| --- | --- |
| 1140883066 | insulin product |
| 1140884600 | metformin |
| 1140874686 | glucophage 500mg tablet |
| 1140857494 | glibornuride |
| 1140857496 | glutril 25mg tablet |
| 1140857500 | glymidine |
| 1140857502 | gondafon 500mg tablet |
| 1140857508 | glucotard 5g/sachet mini-tablet |
| 1140857586 | dimelor 500mg tablet |
| 1140857584 | acetohexamide |
| 1140868902 | acarbose |
| 1140868908 | glucobay 50mg tablet |
| 1140874646 | glipizide |
| 1140874650 | glibenese 5mg tablet |
| 1140874652 | minodiab 2.5mg tablet |
| 1140874658 | gliquidone |
| 1140874664 | tolazamide |
| 1140874674 | tolbutamide |
| 1140874678 | glyconon 500mg tablet |
| 1140874706 | chlorpropamide |
| 1140874718 | glibenclamide |
| 1140874720 | glibenclamide + metformin |
| 1140874728 | euglucon 2.5mg tablet |
| 1140874736 | diabetamide 2.5mg tablet |
| 1140874744 | gliclazide |
| 1140874746 | diamicron 80mg tablet |
| 1141152590 | glimepiride |
| 1141157284 | glipizide product |
| 1141168660 | repaglinide |
| 1141168668 | novonorm 0.5mg tablet |
| 1141171646 | pioglitazone |
| 1141171652 | actos 15mg tablet |
| 1141173882 | nateglinide |
| 1141177600 | rosiglitazone |
| 1141177606 | avandia 4mg tablet |
| 1141189090 | rosiglitazone 1mg / metformin 500mg tablet |
| 1141189094 | avandamet 1mg / 500mg tablet |
